# The effect of TIM1^+^ Breg cells in liver ischemia-reperfusion injury

**DOI:** 10.1038/s41419-025-07446-x

**Published:** 2025-03-12

**Authors:** Yu Zhang, Cheng Zhang, Beng Yang, Chuanhui Peng, Jie Zhou, Shenli Ren, Zhenhua Hu

**Affiliations:** 1 Division of Hepatobiliary and Pancreatic Surgery, Department of Surgery, The First Affiliated Hospital, Zhejiang University School of Medicine, Hangzhou, Zhejiang China; 2 Division of Hepatobiliary and Pancreatic Surgery, Department of Surgery, Zhejiang University School of Medicine Fourth Affiliated Hospital, Yiwu, Zhejiang China

**Keywords:** Cell biology, Diseases

## Abstract

Liver transplantation is the only effective method for end-stage liver disease; however, liver ischemia reperfusion injury (IRI) seriously affects donor liver function after liver transplantation. IRI is a pathophysiological process in which organ damage is aggravated after the blood flow and oxygen supply of ischemic organ tissues are restored. It combines the two stages of hypoxic cell stress triggered by ischemia and inflammation-mediated reperfusion injury. Herein, we studied the protective effect and mechanism of the anti-T cell Ig and mucin domain (TIM1) monoclonal antibody, RMT1-10, on hepatic cell injury induced by IRI. First, a liver IRI model was established in vivo. HE, TEM, and Tunel were used to detect liver tissue injury, changes in the liver ultrastructure and liver cell apoptosis, respectively. ELISA were performed to determine the levels of ALT, AST, MDA, GSH, and related inflammatory factors. We found that RMT1-10 could significantly reduce liver injury. Flow cytometry results showed that the number of TIM1^+^ regulatory B cells (Bregs) in the IRI liver increased briefly, while pretreatment with RMT1-10 could increase the number of TIM1^+^ Bregs and interleukin-10 (IL-10) secretion in liver IRI model mice, thus playing a protective role in liver reperfusion. When Anti-CD20 was used to remove B cells, RMT1-10 had a reduced effect on liver IRI. Previous data showed that the number of T helper 1 cells (Th1:CD4^+^; CD8^+^) increased significantly after IRI. RMT1-10 inhibited Th1 cells; however, it significantly activated regulatory T cells. Sequencing analysis showed that RMT1-10 could significantly downregulate the expression of nuclear factor-kappa B (NF-κB) pathway-related genes induced by IRI. These results suggested that RMT1-10 could promote the maturation of B cells through an atypical NF-κB pathway, thereby increasing the number of TIM1^+^ Bregs and associated IL-10 secretion to regulate the inflammatory response, thereby protecting against liver IRI.

## Introduction

Liver disease resulting in end-organ failure is a growing cause of death. In most cases, the only effective treatment is liver transplantation [1]. However, liver transplantation is a complex undertaking and its success depends on many factors, with liver damage caused by hot ischemia during organ retrieval and prolonged cold storage before transplantation often leading to primary graft dysfunction, predisposition to late chronic rejection, and a shortage of donor organs [2]. In particular, liver transplantation is at risk of ischemia reperfusion injury (IRI), which significantly affects liver function after transplantation. Ischemia reperfusion injury is a pathophysiological process in which hypoxic organ injury is aggravated after blood flow and oxygen supply of ischemic tissue are restored. Tissue injury caused by IRI is a combination of hypoxic cell stress triggered by ischemia and inflammation-mediated reperfusion injury. The essence of IRI is inflammation [3]. However, the role of inflammation and the immune response in the outcome of IRI has not been fully determined, thus requiring further exploration.

Immune cells participate in the body’s immune response and protect the organismal health by exerting their roles under physiological conditions, but can aggravate tissue damage under certain pathological conditions [4]. B cells not only directly mediate the chemotaxis of other immune cells enriched in immune damage and inflammation, but also act as antigen-presenting cells to activate adaptive immunity [5]. These B cells with immunoregulatory functions have been defined as regulatory B cells (Bregs). These Bregs act as negative regulators of the immune system by producing IL-10 and other anti-inflammatory mediators, as well as by suppressing the inflammatory response through direct cellular contact [6]. In in vivo experiments, studies found that Bregs inhibited allergic airway inflammation, promoted transplant tolerance, and improved experimental autoimmune diseases [7–9]. It has been reported that recruitment of Bregs to the heart under myocardial IRI leads to increased expression of immunoregulatory mediators [10]. In renal IRI, treatment with an anti-CD45RB antibody could attenuate renal IRI via induction of Bregs [11]. However, the role of Bregs in hepatic IRI has not been reported.

T cell Ig and mucin domain (TIM1), a transmembrane glycoprotein member of the TIM family of phosphatidylserine receptors on the surface of human and mouse cells, binds directly to members of phosphotyrosine-dependent intracellular signaling pathways and provides co-stimulatory signals for T cell activation [12]. In the immune system, TIM1 was initially found to be expressed in T cells and dendritic cells, in which it plays an important role in regulating vital cellular functions. Recently, TIM1 was found to be mainly expressed on Bregs, acting as a marker of Bregs. The activity of Bregs is mainly attributed to the expression of interleukin-10 (IL-10) [13]. The low-affinity anti-TIM1 monoclonal antibody (RMT1-10) binds to TIM1 and promotes immune tolerance through IL-10-expressing B cells, increases the number of TIM1^+^ B cells and the percentage of TIM1^+^ B cells expressing IL-10 and IL-4, and preserves regulatory T cells; however, it inhibited CD4^+^ T helper 1 (Th1) cells [14, 15]. In a study of atherosclerosis, RMT1-10 could specifically expand TIM1^+^ IgM^+^ B1a cells and reduce the number of CD4^+^ CD8^+^ T cells, thereby reducing arterial inflammation and inhibiting the progression and development of atherosclerosis [14]. These studies suggested that TIM1 is critical to regulate the function of Bregs, suppress inflammatory responses, and maintain self-tolerance. Furthermore, in a mouse liver IRI model, RMT1-10 was used as a pretreatment to prevent liver IRI, which significantly inhibited hepatocyte apoptosis [16]. A previous study also found that RMT1-10 treatment could effectively inhibit the inflammatory response of cerebral IRI, thereby reducing brain injury [17]. However, the role of TIM1^+^ Bregs in hepatic IRI needs to be further studied.

Herein, we report the effect of RMT1-10 on liver IRI and its underlying mechanism. Our findings provide strategies to develop TIM1^+^ Breg cell therapies and identify potential therapeutic targets to modulate TIM1^+^ Breg function in liver IRI.

## Results

### RMT1-10 treatment reduced liver IRI injury

**Fig. 1:**
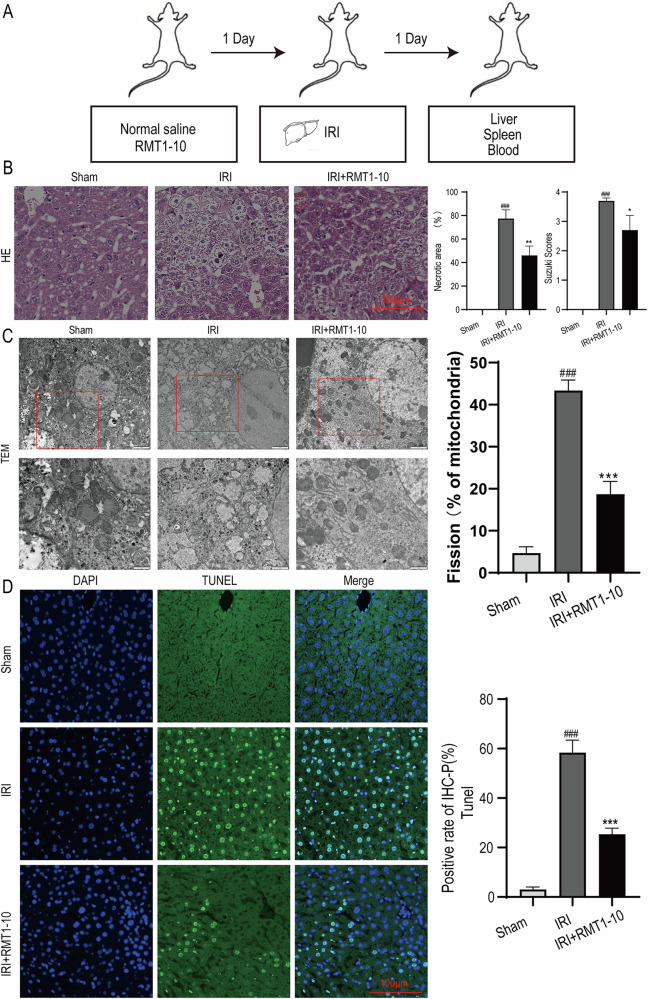
RMT1-10 treatment reduced liver IRI injury. **A** The source of the mice and an image of the liver IRI model. **B** HE was used to analyze the extent of liver damage (*n* = 3). **C** Liver tissue ultrastructure was assessed using TEM analysis (*n* = 3). **D** TUNEL staining to determine liver cell apoptosis. ###*P* < 0.001 *vs*. Sham; ***P < 0.001 vs. IRI (*n* = 3).

First, we established the liver IRI model. The source of mice and an image of the IRI liver were taken from https://www.bioon.com.cn/news/showarticle.asp?newsid=72124 and https://xsj.699pic.com/tupian/16i428.html and are shown in Fig. 1A. Next, hematoxylin and eosin (HE) staining was used to detect liver damage after treatment with or without **RMT1-10** under the IRI conditions (Fig. 1B). Liver damage was aggravated in the IRI group in comparison with the Sham group; however, RMT1-10 treatment could alleviate this damage. Evaluation of liver tissue ultrastructure using TEM showed that compared with that in the sham operation group, the IRI group showed swelling, cell membrane rupture, porosity, incomplete mitochondrial and lysosomal structures, reduced ridge count, and disintegrated subcellular organelles. However, after treatment with RMT1-10, the swelling of the cells was reduced, the cell membrane was continuous, and the structure of each organelle was relatively intact (Fig. 1C). Further terminal deoxynucleotidyl transferase nick-end-labeling (TUNEL) staining showed that the number of apoptotic cells was increased under IRI conditions, while RMT1-10 treatment could reduce apoptosis (Fig. 1D). These findings indicated that RMT1-10 could alleviate IRI-induced liver IRI.

### RMT1-10 increased the number of TIM-1^+^Bregs and regulated the expression of inflammatory factors after liver IRI-induction

**Fig. 2:**
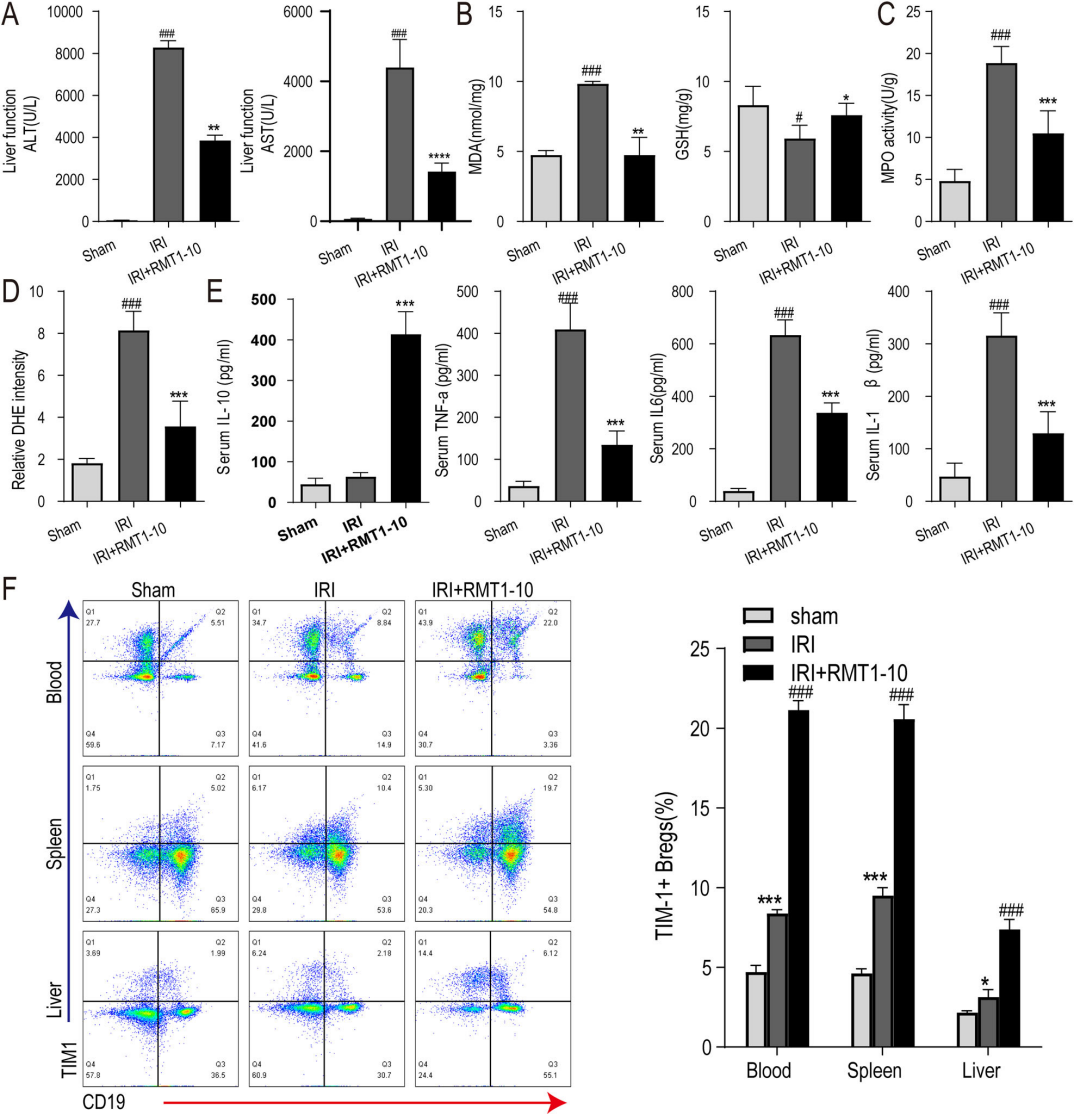
RMT1-10 increased the number of TIM-1^+^Breg cells and regulated the expression of inflammatory factors after liver IRI-induction. **A**–**C** The changes in liver ALT and AST in peripheral blood serum from different groups (sham, IRI, and RMT1010 + IRI). ###*P* < 0.001 vs. Sham; **P < 0.01 vs. IRI. The levels of MDA, GSH, and MPO in liver tissue of the sham, IRI, and RMT1-10 + IRI groups. #*P* < 0.05, ###*P* < 0.001 vs. Sham; *P < 0.05, **P < 0.01, ***P < 0.001 vs. IRI, (*n* = 5). **D** Confirmation of DHE intensity in the sham, IRI, and RMT1-10 + IRI groups. ###*P* < 0.001 vs. Sham; **P < 0.01 vs. IRI, (*n* = 5). **E** Levels of inflammatory factors IL-10, TNFα, IL-6, and IL-1β in peripheral blood, (*n* = 5). **F** The number of TIM-1^+^ Bregs (TIM-1^+^ CD19^+^) in the spleen, heart, and blood was determined using Flow cytometry. *P < 0.05, ***P < 0.001 vs. sham, ###*P* < 0.001 vs. IRI, (*n* = 3).

Next, we investigated the effect of RMT1-10 on liver functional impairment after liver IRI. First, we measured the level of Alanine transaminase (ALT) and Aspartate transaminase (AST) in peripheral blood serum, which showed that IRI upregulated ALT and AST levels, while RMT1-10 treatment downregulated them (Fig. 2A). Then, we detected the level of Malondialdehyde (MDA) and glutathione (GSH) in liver tissue, which indicated that RMT1-10 could inhibit the IRI-induced the upregulation of MDA and increased the level of GSH under IRI (Fig. 2B). Our data further found that the activity of MPO and the intensity of DHE were reduced after treatment with RMT1-10 in liver IRI (Fig. 2C, D). ELISA examination showed that IL-10, TNFα, IL-6 and IL-1β levels were increased in the peripheral blood of the IRI group. RMT1-10 treatment further upregulated IL-10 levels; however, the levels of TNFα, IL-6, and IL-1β were downregulated (Fig. 2E). Flow cytometry showed that the number of TIM1^+^ Bregs (TIM1^+^ CD19^+^) in PBMCs, and spleen and liver immune cells was increased, which were further promoted by RMT1-10 treatment (Fig. 2F).

The diagram showing the IRI model establishment and the gating strategies for TIM1^+^ Bregs in the spleen, liver, and blood are shown in Supplementary Fig. 1A, B. Next, we determined the expression of TIM1 after treatment with or without RMT1-10 in liver IRI, which showed that IRI resulted the upregulation of TIM1, whereas RMT1-10 treatment further promoted the expression of TIM1 (Supplementary Fig. 1C).

#### The protective effect of RMT1-10 on liver IRI was partially reversed by treatment with Anti-CD20

**Fig. 3:**
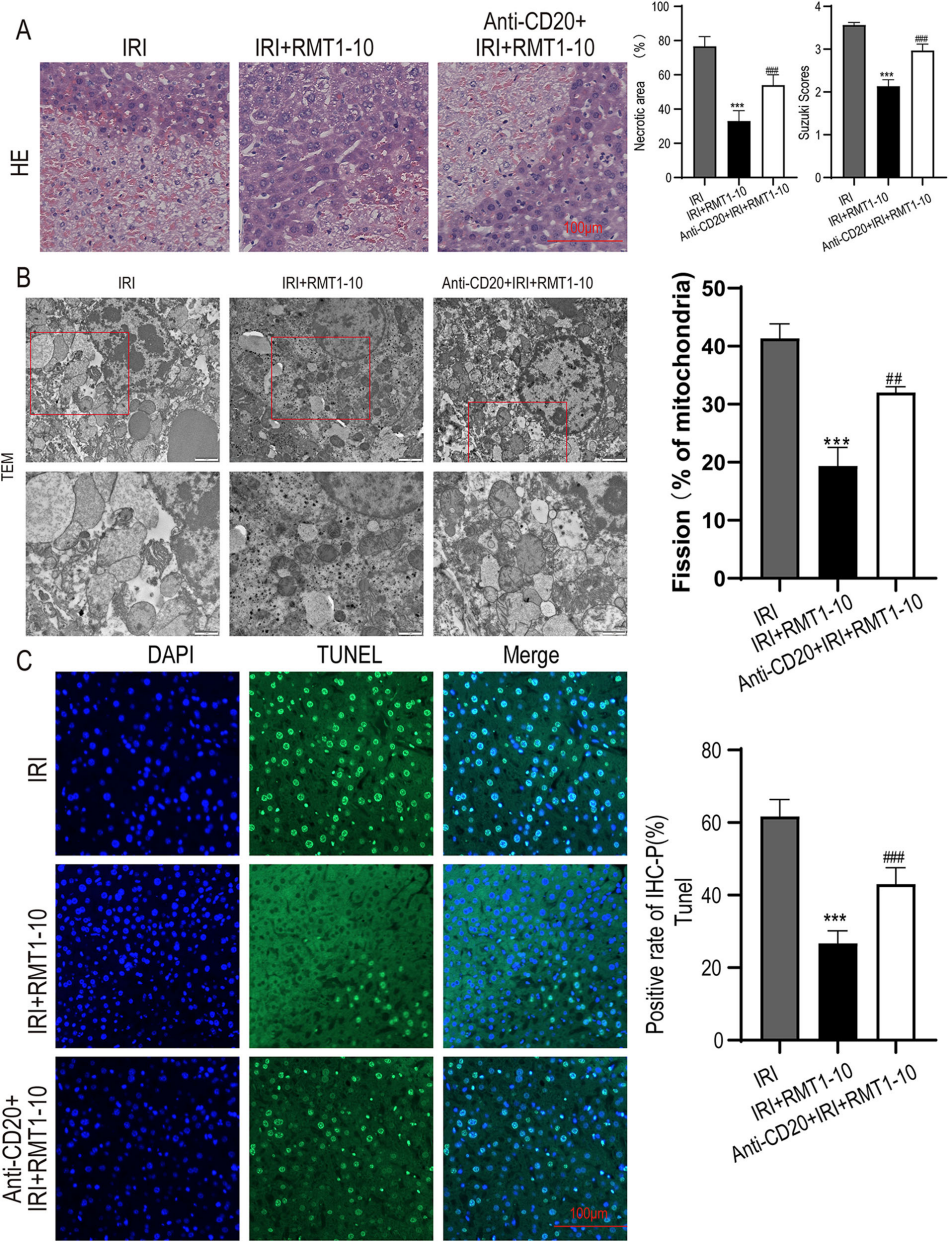
The liver damage alleviated by RMT1-10 was aggravated by treatment with Anti-CD20. **A** HE was used to analyze the extent of liver damage in different groups (IRI, IRI + RMT1-10, and Anti-CD20 + IRI + RMT1-10). ###*P* < 0.001 vs. IRI; ***P < 0.001 vs. RMT1-10 + IRI, (*n* = 3). **B** The liver tissue ultrastructure in IRI, IRI + RMT1-10, and Anti-CD20 + IRI + RMT1-10 groups, as assessed using TEM analysis, (*n* = 3). **C** TUNEL staining to determine liver cell apoptosis after treatment with RMT1-10 combined with Anti-CD20. ***P < 0.001 vs. IRI; ###*p* < 0.001 vs. IRI + RMT1-10, (*n* = 3).

To explore whether the protective effect of TIM-1^+^ Bregs in liver IRI acts via inhibition of immune inflammation, we used Anti-CD20 to remove B cells, and then established a liver IRI model, followed by treatment with RMT1-10. The results of HE analysis showed that the reduction in liver damage induced by RMT1-10 was partially reversed by combination with Anti-CD20 (Fig. 3A). Transmission electron microscopy (TEM) analysis of the liver tissue ultrastructure indicated that Anti-CD20 combined with RMT1-10 treatment enhanced liver cell injury (Fig. 3B). Moreover, the number of apoptotic cells was increased after co-treatment with RMT1-10 and Anti-CD20, according to TUNEL staining in comparison with that in the RMT1-10 only group (Fig. 3C). These findings showed that although RMT1-10 could alleviate liver IRI, Anti-CD20 + RMT1-10 treatment aggravated it.

### Anti-CD20 reduces the number of TIM-1^+^Bregs and enhances expression of inflammatory factors after co-treatment with RMT1-10 in liver IRI

**Fig. 4:**
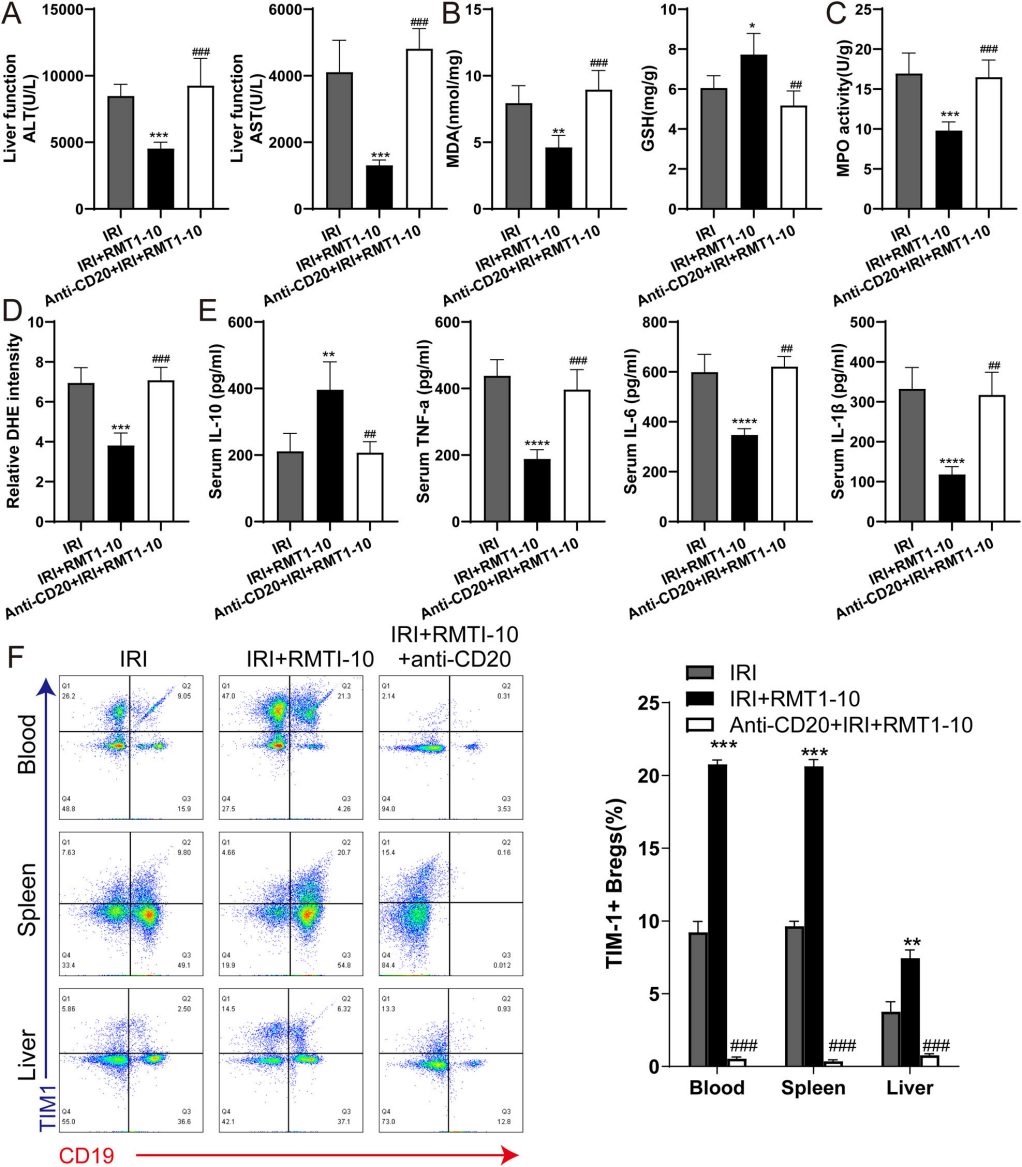
Anti-CD20 reduces the number of TIM-1^+^Bregs and enhances the expression of inflammatory factors after co-treatment with RMT1-10 in liver IRI. **A** The levels of ALT and AST in the peripheral blood serum in the IRI, IRI + RMT1-10, and Anti-CD20 + IRI + RMT1-10 groups.***P < 0.001 vs. IRI; ###*P* < 0.001 vs. IRI + RMT1-10, (*n* = 5). **B**, **C** The levels of MDA, GSH and MPO in liver tissue from the IRI, IRI + RMT1-10, and Anti-CD20 + RMT1-10 + IRI groups. *P < 0.05, **P < 0.01, ***P < 0.001 vs. IRI; ##*P* < 0.01, ###*P* < 0.001 vs. IRI + RMT1-10, (*n* = 5). **D** Confirmation of the DHE intensity in the IRI, IRI + RMT1-10, and Anti-CD20 + RMT1-10 + IRI groups. ***P < 0.001 vs. IRI; ###*P* < 0.001 vs. IRI + RMT1-10, (*n* = 5). **E** Levels of inflammatory factors IL-10, TNFα, IL-6 and IL-1β in the peripheral blood from the IRI, IRI + RMT1-10, and Anti-CD20 + RMT1-10 + IRI groups. ***P < 0.001 vs. IRI; ##*P* < 0.01 ###*p* < 0.001 vs. IRI + RMT1-10, (*n* = 5). **F** The number of TIM-1^+^ Bregs (TIM-1^+^ CD19^+^) was determined using Flow cytometry. **P < 0.01,***P < 0.001 vs. IRI; ###*P* < 0.001 vs. IRI + RMT1-10, (*n* = 3).

To further reveal the effect of Anti-CD20 on the inflammatory response, we measured the levels of ALT, AST, MDA, and GSH in peripheral blood serum or liver tissue. The results indicated that RMT1-10 treatment could reduce the level of ALT, AST, and MDA, and increase the GSH level, whereas RMT1-10 combined with Anti-CD20 increased ALT, AST and MDA, and reduced GSH levels (Fig. 4A, B). Anti-CD20 combined with RMT1-10 also upregulated the activity of Myeloperoxidase (MPO) and the intensity of dihydroethidium (DHE) compared with that induced by RMT1-10 alone under liver IRI conditions (Fig. 4C, D). The upregulation of IL-10 by RMT1-10 was reversed after co-treatment with Anti-CD20, and the downregulation of TNFα, IL-6, and IL-1β levels was also reversed after treatment by RMT1-10 plus with Anti-CD20 (Fig. 4E). Flow cytometry further determined the number of TIM1^+^ Bregs (TIM-1^+^ CD19^+^), showing that compared with the that in the IRI + RMT1-10 group, the number of TIM1^+^ Bregs cells was decreased after co-treatment with RMT1-10 and Anti-CD20 under liver IRI conditions (Fig. 4F).

### RMT1-10 treatment increases TIM1^+^ Bregs and IL-10 secretion in vitro

**Fig. 5:**
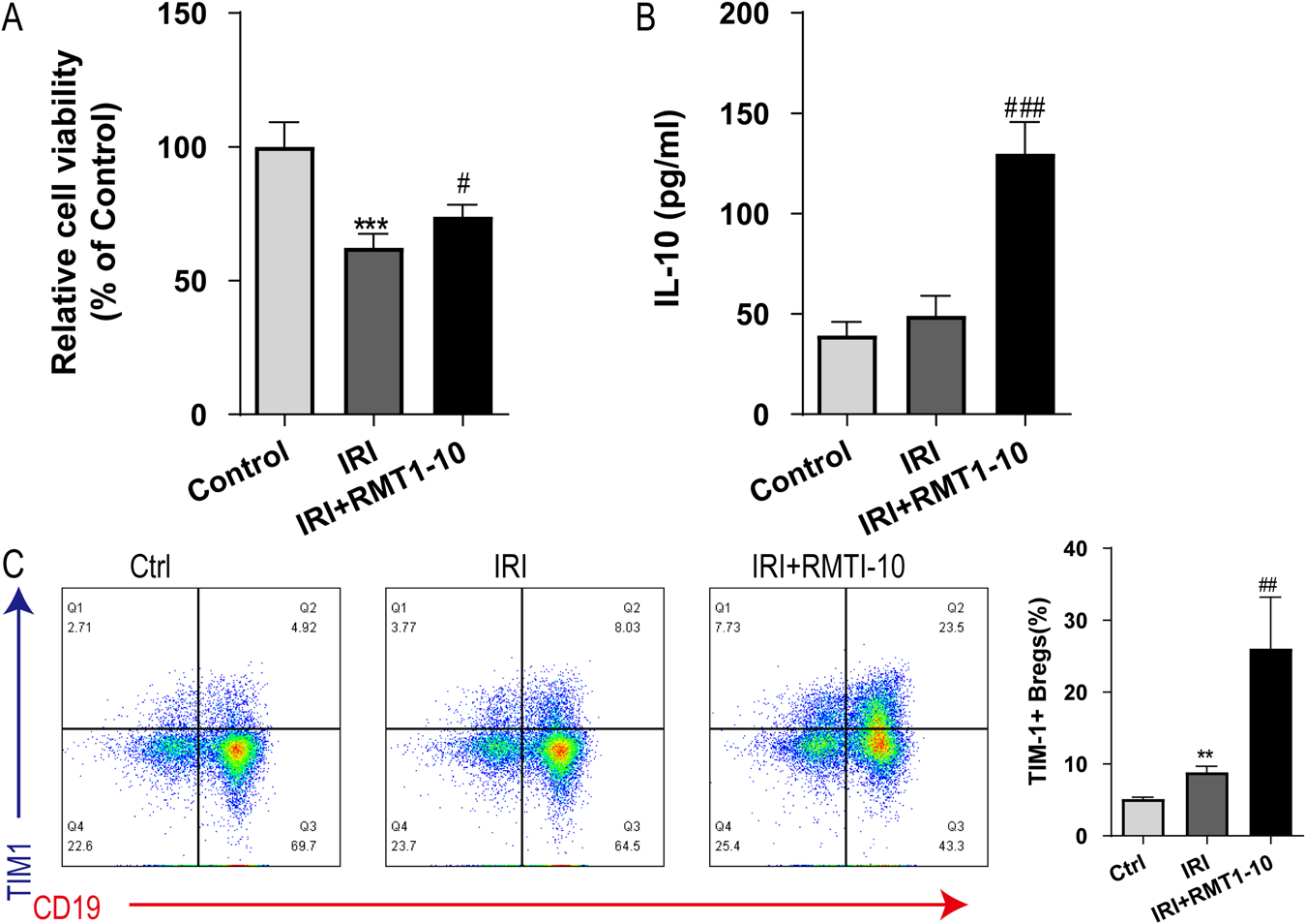
RMT1-10 treatment increases TIM-1^+^ Breg numbers and IL-10 secretion in vitro. **A** CCK8 assay to determine the liver cell viability in the Control, IRI, and IRI + RMT1-10 groups. ***P < 0.001 vs. Control, #*P* < 0.05 vs. IRI, (*n* = 6). **B** The expression of IL-10 in supernatant, as detected by ELISA. **P < 0.01 vs. Control, ###*P* < 0.001 vs. IRI, (*n* = 3). **C** Flow cytometry examination of the number of TIM-1^+^ Bregs (TIM-1^+^ CD19^+^). **P < 0.01 vs. Control, ##*P* < 0.01 vs. IRI, (*n* = 3).

To further explore the protective mechanism of RMT1-10 in vitro, we constructed a co-culture system of immune cells (TIM-1^+^ Bregs and CD4^+^ naïve T cells; TIM-1^+^ Bregs and CD8^+^ naïve T cells) and liver cell, these cells were extracted from mice in the sham and IRI group. After treatment with RMT1-10, we observed changes in cell activity, as well as changes in the amount of IL-10 and TIM1^+^Bregs. The results showed that RMT1-10 treatment could reverse the decrease in cell viability caused by IRI (Fig. 5A). Although IRI resulted in slight upregulation of IL-10, RMT1-10 treatment markedly increased the expression of IL-10 (Fig. 5B), a trend that was also reflected in the number of TIM1^+^ Bregs (Fig. 5C). These findings indicated that RMT1-10 could promote the secretion of IL-10 by TIM1^+^ Bregs.

The homologous interaction between Breg cells and T cells is considered to control the induction of Tregs [18–20]. Flow cytometry determined that the differentiation of Th1 (CD4^+^ T and CD8^+^ T) subsets was active after IRI; however, RMT1-10 could inhibit the differentiation of Th1 (CD4^+^ T and CD8^+^ T) cells (Supplementary Fig. 2A). After RMT1-10 treatment, Tregs (CD4^+^CD25^+^Foxp3^+^) were significantly activated (Supplementary Fig. 2B). When B cells were removed by treatment with Anti-CD20 antibodies, RMT1-10 treatment did not increase and activate Tregs (CD4^+^CD25^+^Foxp3^+^) after IRI (Supplementary Fig. 2C). These findings indicated that TIM1^+^ Bregs promoted the secretion of the immunomodulatory cytokine IL-10, thus further regulating Tregs, inhibiting the inflammatory response, and alleviating liver IRI.

### The molecular mechanism by which RMT1-10 promotes the proliferation of TIM1+ Bregs and the secretion of IL-10

**Fig. 6:**
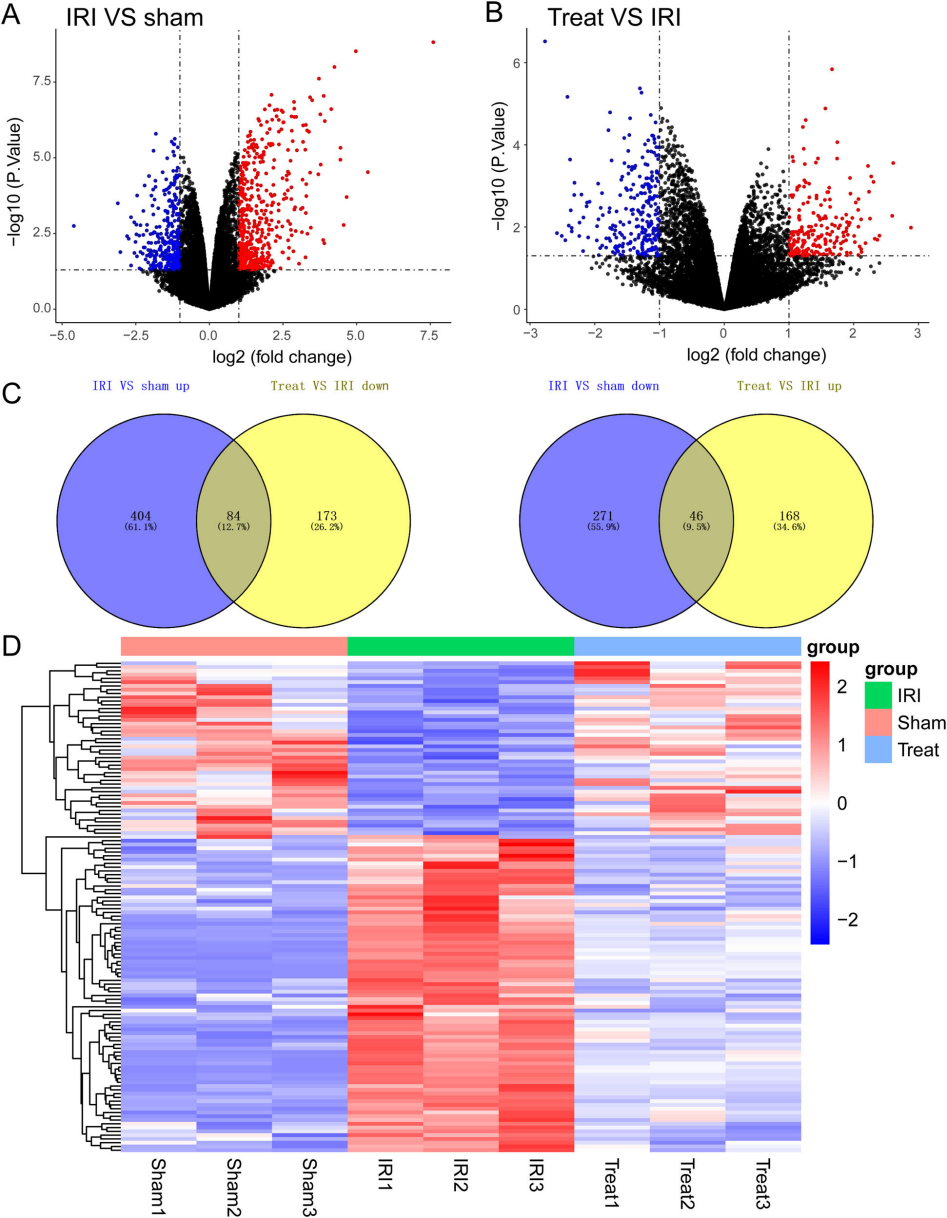
The molecular mechanism by which RMT1-10 promotes the proliferation of TIM-1^+^ Bregs and the secretion of IL-10. **A**, **B** The expression levels of genes from different groups were analyzed using volcano map, (*n* = 3). **C** Venn diagrams of the upregulated and downregulated genes in the sham, IRI, and IRI + RMT1-10 groups. **D** Heat map analysis of the screened genes.

To further investigate the signaling pathway through which RMT1-10 increases the number of TIM1^+^ Bregs cells and the secretion of IL-10, we carried out sequencing analysis for the sham group, liver IRI group, and RMT1-10 + IRI group. After further analysis, we constructed a volcano plot showing a map of the upregulated and downregulated differentially expressed genes between the sham and IRI groups, and between the IRI and IRI + RMT1-10 groups (Fig. 6A, B). Intersection analysis of the downregulated genes between the sham and IRI groups and the upregulated genes between the IRI and IRI + RMT1-10 identified 84 genes. Intersection analysis of the upregulated genes between the sham and IRI groups and the downregulated genes between the IRI and IRI + RMT1-10 groups identified 46 genes (Fig. 6C). This information was processed and presented as a heat map (Fig. 6D).

### RMT1-10 protects liver IRI via NF-κB pathway

**Fig. 7:**
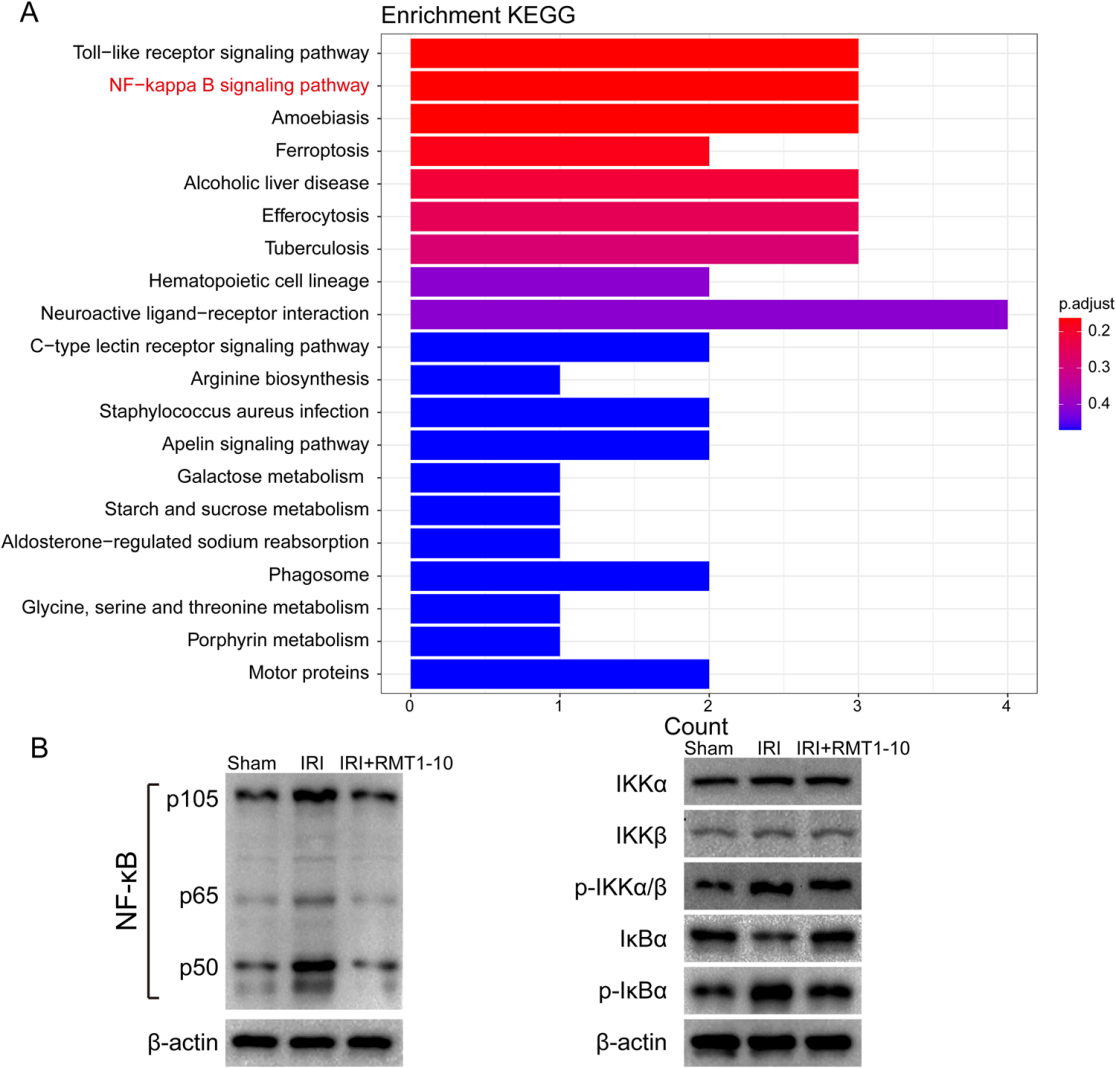
RMT1-10 protects against liver IRI via the Nuclear factor kappa B pathway. **A** KEGG enrichment analysis of the differentially expressed genes shown in Fig. 6. **B** Western blotting detection the levels of p-IkBα, p-IkBβ, and p-IKKBα/β.

Next we used Kyoto Encyclopedia of Genes and Genomes (KEGG) enrichment analysis to analyze the above differentially expressed genes, which showed that these genes were enriched in many signaling pathways. We selected the nuclear factor kappa B (NF-κB) Signaling pathway for further analysis (Fig. 7A). Furthermore, we detected NF-κB pathway-related protein levels, which showed that phosphorylated (p)-IkBα, p-IkBβ, and p-IKKBα/β levels were upregulated after IRI, while RMT1-10 treatment downregulated their levels (Fig. 7B). These data indicated that RMT1-10 could promote the maturation of B cells through an atypical NF-κB pathway to increase the number of TIM1^+^ Bregs, thereby increasing the secretion of IL-10 to regulate the inflammatory response, thus protecting against liver IRI.

## Discussion

In the present study, we determined the protection provided by RMT1-10 against liver IRI. The results indicated that RMT1-10 treatment could increase the number of TIM1^+^ Bregs and IL-10 secretion, which further regulated the expression of inflammatory factors, thereby alleviating liver IRI. The transmembrane glycoprotein TIM1 regulates immune responses and thus plays an important role in liver IRI. TIM1 attenuates the protection of ischemic preconditioning for ischemia reperfusion injury in liver transplantation [16]. In addition, TIM1 regulates T cell activation and affects macrophage function to protect against liver IRI [21]. It has been reported that RMT1-10 can induce an increase in the number of TIM1^+^ Bregs and increase the secretion of cytokine IL-10 [14, 22]. However, TIM1 defects or mutations will reduce the production of IL-10 in B cells, and increase the levels of pro-inflammatory cytokines in T cells, such as IL-1 and IL-6, IL-17 and interferon gamma (IFNγ), and inhibition of Foxp3^+^ regulatory T cells (Tregs) and type 1 regulatory T cells (Tr1) [22, 23]. In mice without TIM1 mutation, Bregs showed a progressive loss of the ability to produce IL-10 and severe multi-organ tissue inflammation with age [13]. Older mice with TIM1 mutation showed decreased IL-10 secretion from B cells, spontaneous autoimmunity associated with T cell overactivity, increased IFNγ production, and elevated serum Ig and autoantibody levels [24]. Herein, we isolated mouse spleen B cells and found that RMT1-10 could significantly upregulate the number of TIM1^+^ positive cells among spleen B cells and the level of IL-10 in the cell supernatant.

Bregs are found in mice and humans, which participate in downregulating the inflammation associated with many pathological processes, including autoimmune diseases, transplant rejection, anti-tumor response, and infection [9]. It has been reported that Bregs prevent the induction of autoimmune responses and inhibit excessive inflammation in autoimmune diseases by promoting the differentiation of regulatory T cells (Tregs) and inhibiting T-helper 1 (Th1) and Th17 inflammatory cells [25]. Their ability to produce anti-inflammatory cytokines, such as IL-10, transforming growth factor beta (TGFβ), and IL-35, as well as their ability to induce Tregs, is thought to underpin their regulatory potential. TGFβ, as an inhibitory molecule of Bregs, has multiple roles in adaptive immunity, especially in regulating CD4^+^ and B cell responses [26]. IL-35 is an anti-inflammatory cytokine that is believed to be produced by Tregs, which can inhibit autoimmune diseases, improve immune tolerance by inducing Treg proliferation, inhibit Th1/Th17 cells, and upregulate Foxp3^+^Treg-related responses [27, 28]. As a result, Bregs have the ability to target many immune system cells, thus playing a pleiotropic role in suppressing the immune response [29]. In our study, detection of IL-10 in peripheral blood and liver IRI tissues of mice showed that compared with the normal group, the expression of IL-10 was partially upregulated after IRI; however, the difference was not significant, while the expression of IL-10 was significantly increased after treatment with RMT1-10. These results suggested that RMT1-10 could promote IL-10 secretion in TIM1^+^ Bregs.

B-cell-mediated expression of IL-10 inhibits the expression of pro-inflammatory cytokines in CD4 + T cells (including TNFα, IFNγ, and IL-17), inhibits the cytotoxicity of CD8^+^ T cells, and promotes the differentiation of Tregs [25, 30]. However, a lack of IL-10^+^ Bregs cells will further aggravate the development of various inflammatory immune diseases [31]. Earlier studies found that in genetically modified mice that lacked B cells (specifically the B cells that produce IL-10), defects in the development and function of Bregs led to chronic inflammation [32]. However, when B cells were introduced into the cerebral cortex of a B-cell-deficient mouse model of cerebral IRI induced by middle cerebral artery occlusion, inflammation and infarct volumes were significantly reduced compared with wild-type controls [33]. The homologous interactions between Bregs and T cells are thought to control the induction of Tregs [34–36]. The importance of B cells in maintaining Tregs was proposed in earlier studies, which showed significant reductions in Tregs in mice lacking B cells [37, 38]. Mice with B-cell-specific IL-10 deficiency also showed Treg deficiency, which was associated with the growth of pro-inflammatory T cells after autoimmune induction [39, 40]. Our findings showed that the protective effect of RMT1-10 disappeared after Anti-CD20 and RMT1-10 co-treatment, which indicated that RMT1-10 plays an important role in B cells under liver IRI conditions. Furthermore, Th1 (CD4^+^CD8^+^) cell numbers increased significantly after liver IRI, whereas their numbers decreased under RMT1-10 treatment, while the number of Treg cells (CD4^+^CD25^+^Foxp3^+^) was significantly increased.

Nuclear factor kappa B comprises a family of transcription factors that regulate the expression of growth factors, cytokines, chemokines, adhesion molecules, and inhibitors of apoptosis [41]. More important for this study, NF-κB regulates B cell growth and survival. Signaling pathways of NF-κB can be divided into typical and atypical types, in which the typical pathway regulates cell proliferation, immune function, and pro-inflammatory responses, while the atypical type leads to B-cell maturation and lymphoid organ formation [42, 43]. Activation of NF-κB affects cell growth and programmed cell death in liver IRI [44]. It has been reported that anti-TIM1 antibody treatment could inhibit NF-κB signaling to improve the hepatocellular function [21], which was consistent with the results of the present study. Sequencing analysis of the changes of in gene expression and signaling among the Sham group, IRI group, and RMT1-10 + IRI group showed that the expression levels of NF-κB pathway-related genes were upregulated by IRI, but downregulated by RMT1-10 treatment.

## Conclusions

In summary, this study demonstrated that RMT1-10 promotes the maturation of B cells through an atypical NF-κB pathway, thereby increasing the number of TIM1^+^ Bregs, which secrete IL-10 to regulate the inflammatory response, and thereby protecting against liver IRI.

## Materials and methods

### Animals

Male wild-type C57BL/6 mice aged 6–8 weeks (weighing 22 to 32 g) were fed under specific pathogen free conditions. The feeding conditions were a temperature range of 20–22 °C, a humidity range of 50–55%, and a 12 h light and dark cycle. All the experimental animals ate and drank freely. Mouse livers were taken as donor organs and stored in University of Wisconsin (UW) solution at 4 °C for 20 h. Other mice were used as recipients for orthotopic liver transplantation. The survival of the mice in each group was observed after surgery, and the mice were sacrificed at different time points. Serum samples and liver tissues were collected.

### Liver IRI model

First, we established a hepatic IRI model. A total of 24 C57BL/6 J mice were randomly divided into four groups (*n* = 6): Sham, IRI, IRI + RMT1-10 (500 µg anti-mouse TIM-1 (BioXCell, Lebanon, NH, USA) i.p. on day 1 before liver IRI, and 300 µg on days 0 and 1, IRI + RMT1-10+ Anti-CD20 (BioXCell, 250 μg/200 μl phosphate buffered saline (PBS)).Sterile anti–mouse CD20 and isotype control mAbs were injected through lateral tail veins on day 14 and 7 before liver IRI. Briefly, mice were fasted for 12 h before surgery, followed by1% isoflurane anesthesia while lying supine. A median incision in the upper abdomen was made to separate the perihepatic ligaments, expose the first hepatic portal, free the middle lobe of the liver, and perform the Glisson system in the left lobe, followed by clamping with a non-invasive vascular clamp. This resulted in 70% liver ischemia, and the corresponding liver lobe turned from bright to pale red after blocking, indicating successful establishment of liver ischemia. Ninety minutes later, the vascular clamp was removed to restore blood flow, followed by reperfusion for 6 h and 12 h. In the sham operation group, only the first hepatic portal was free after laparotomy without blocking the blood flow. After 6 h and 12 h of reperfusion, blood was collected from the eyeball, the supernatant was obtained by centrifugation, and stored at −80 °C. The mice were then sacrificed and left and middle lobe liver tissue samples were taken.

### Terminal deoxynucleotidyl transferase nick-end-labeling (TUNEL) staining

The liver tissue was cut into 0.5 cm^3^ pieces, fixed with 4% paraformaldehyde phosphate, dehydrated, impregnated with wax, embedded, and sectioned at a thickness of 10 μm. Later, apoptosis was determined using an in situ cell death detection kit (Roche, Basel, Switzerland) in accordance with the manufacturer’s instructions. Cell apoptosis was observed under a light microscope. Normal liver nuclei were colored blue and apoptotic nuclei were brown. The number of liver nuclei was not less than 500, and the apoptosis rate was calculated using Image-Pro Plus v.6.0 software (Media Cybernetics, Rockville, MD, USA).

### Hematoxylin and eosin (HE) staining

The middle lobe of the liver was taken, fixed in 4% paraformaldehyde for 24 h, embedded in paraffin, and sectioned in a graded ethanol series. The sections were subjected to HE staining, and the liver tissue injury was observed under an optical microscope (Olympus, Tokyo, Japan).

### Enzyme-linked immunosorbent assay (ELISA)

The levels of Alanine transaminase (ALT), Aspartate transaminase (AST), glutathione (GSH), Myeloperoxidase (MPO), Malondialdehyde (MDA), dihydroethidium (DHE), IL-10, tumor necrosis factor alpha (TNFα), IL-6, IL-1β were determined by ELISA according to the manufacturers’ instructions (R&D Systems, Minneapolis, MN, USA).

### Transmission electron microscopy (TEM)

Transmission electron microscopy was used to observe the ultrastructure of liver cells. Briefly, liver tissue was cut into small pieces of 1 mm^3^ and fixed with glutaraldehyde phosphate buffer, further fixed with 1% osmic acid, dehydrated with conventional ethanol, impregnated with propylene oxide, and embedded in resin. Ultrathin sections of 50–70 nm were prepared, and stained with lead and uranium. The ultrastructure of liver cells was then observed using a Transmission electron microscope (Olympus).

### Isolation and identification of immune cells and peripheral blood mononuclear cells (PBMCs)

The CD4^+^ naïve T (CD8^+^ naïve T) cells were separated from mouse spleens. The mouse spleens were excised, cut into small pieces, digested, and centrifuged. The supernatant was collected as the liver cell suspension. The pellet was suspended in 3 ml of PBS, the lymphocyte separation solution of Biologic was centrifuged with 300 g for 30 min, and then the layers were obviously stratified, the middle white membrane was washed twice, the antibody flow was incubated, and the test was carried out on the machine. The same method was used to extract immune cells (TIM1^+^ Bregs B cells) from mouse PBMCs.

### Flow cytometry analysis

Flow cytometry was performed to determine the numbers of TIM1^+^ Bregs (TIM1^+^ CD19^+^) and Tregs (CD4^+^ CD25^+^ Foxp3^+^). Isolated cells were treated with anti-TIM1, anti-CD19, anti-CD4, anti-CD25, anti-forkhead box P3 (Foxp3) antibodies (Biolegend, San Diego, CA, USA). The data were acquired using a Cytek Aurora 3000 spectral flow cytometer (Cytek, Fremont, CA, USA), and analyzed using FlowJo software v10.7.1 (Treestar Inc., Ashland, OR, USA).

### Co-culture of immune cells

CD4^+^ naïve T cells (CD8^+^ naïve T) and TIM-1^+^ Bregs (TIM-1^+^ CD19^+^ B cells) were sorted from among the mouse PBMC using flow cytometry. Flow cytometry data were acquired using a Cytek Aurora 3000 spectral flow cytometer (Cytek, Fremont, CA, USA), and analyzed using FlowJo software v10.7.1 (Treestar Inc., Ashland, OR, USA). CD4^+^ naïve T cells were co-stimulated with anti-CD3 (5 µg/ml)/anti CD28 (5 µg/ml) antibodies (1 h) and placed in the lower chamber of a Transwell plate. TIM-1^+^ Bregs (treated/not treated with anti-IL-10 neutralizing antibody (10 µg/ml) or/and anti-TGF-β (10 µg/ml)) were placed in the upper chamber for 3 days. Thereafter, cells from the lower chamber were harvested and analyzed for Tregs (CD4^+^ CD25^+^ Foxp3^+^).

### Western blotting analysis

First, cells from different groups were processed using cell lysis buffer (Beyotime, China). Then, 40 μg of protein were separated using 10% sodium dodecyl sulfate-polyacrylamide gel electrophoresis (SDS-PAGE), followed by transfer to polyvinylidene fluoride (PVDF) membranes (Millipore, Billerica, MA, USA). The membranes were then blocked using Tris-buffered saline-Tween20 (TBS-T) including 5% bovine serum albumin for 2 h at 37 °C. After washing with TBS-T three times, the membranes were incubated overnight with primary antibodies recognizing I-kappa-B kinase-alpha (IKKα), phosphorylated (p)-IKKα, inhibitor of nuclear factor kappa B kinase subunit beta (IKKβ), p-IKKβ, NF-Kappa-B Inhibitor Alpha (IκBα), and p-IκBα (Abcam, Cambridge, MA, USA; diluted 1:1000 in TBS-T) at 4 °C. Following further washes with TBS-T, the membranes were incubated with horseradish peroxidase (HRP)-conjugated secondary antibodies (Abcam) for 2 h at 37 °C. Glyceraldehyde-3-phosphate dehydrogenase (GAPDH) was used as an internal control. The protein bands were detected using chemiluminescence (GE Healthcare, Piscataway, NJ, USA).

### Quantitative real-time reverse transcription PCR (qRT-PCR)

Total RNA was extracted from all samples using the TRIzol reagent (Thermo Fisher Scientific, Waltham, MA, USA) and the RNA was reverse-transcribed into cDNA using a Prime-Script RT reagent kit (Takara, Dalian, China). The qPCR step of the qRT-PCR protocol was carried out using the cDNA as the template and 2× SYBR Green PCR Master Mix (Takara) according to the manufacturer’s instructions in an ABI 7500 real-time PCR system (Applied Biosystems, Foster City, CA, USA). The relative gene expression levels were analyzed using the comparative 2^-ΔΔCt^ method. All the experiments were repeated at least three times. The following primers were used:

IL-10

Forward: 5ʹ-GAAGACCCTCAGGATGCGGC -3ʹ;

Reverse: 5ʹ-AAGAGACCCGACACCGGACA -3ʹ

TNF-α

Forward: 5ʹ- GCCAACCAGGCAGGTTCTGT -3ʹ;

Reverse: 5ʹ- TAGGCACCGCCTGGAGTTCT -3ʹ

IL-6

Forward: 5ʹ- GCCCTCTGGCGGAGCTATTG -3ʹ;

Reverse: 5ʹ- AAGGCCGTGGTTGTCACCAG -3ʹ

### Sequencing analysis

Sequencing analysis of mRNA from Splenic B cells was conducted by NOVO GENE Co., Ltd. (Beijing, China). Samples were obtained from mouse spleen cells (with red blood cells removed) in the sham surgery group, the liver IRI group, and the RMT1-10 + IRI group. We then processed the samples and sent to NOVO GENE for sequencing and analysis. NOVO GENE used an Agilent 2100 bioanalyzer (Agilent, Santa Clara, CA, USA), which accurately detects RNA integrity and total volume, and builds libraries for Illumina sequencing (Illumina Inc., San Diego, CA, USA). An index of the reference genome was then constructed using HISAT2 v2.0.5, and paired end clean reads were compared with the reference genome using HISAT2 v2.0.5, with featureCounts (1.5.0-p3) being used to calculate the readings mapped to each gene. The DESeq2 software (1.20.0) was used to conduct differential expression analysis between the samples from the two groups. According to DESeq2, genes with an adjusted *P* value ≥0.05 were assigned as differentially expressed.

### Gene ontology (GO) and Kyoto Encyclopedia of Genes and Genomes (KEGG) pathway enrichment analysis

Molecular functions and biological processes of the differentially expressed genes were clarified using GO enrichment analysis. The key pathways involving the differentially expressed genes were predicted using KEGG pathway enrichment analysis.

### Statistical analysis

The data are expressed as the mean ± standard deviation (SD). Comparisons between two groups were performed using Student’s *t* test or one-way analysis of variance, followed by Tukey’s post-hoc test to compare multiple groups. Statistical analysis was performed using GraphPad Prism 9.0 software (GraphPad Software, San Diego, CA, USA). Statistical significance was considered at a *P* value < 0.05.

## Supplementary information

Supplementary Figure legends

Supplement Figure 1

Supplement Figure 2

Original WB

## Data Availability

All data supporting the findings of this study are included within the manuscript and its supplementary information files. Raw data and additional materials will be made available by the authors upon reasonable request.
