# Supplementary material for: The effect of TIM1+ Breg cells in liver ischemia-reperfusion injury
Source: Cell Death Dis. 2025 Mar 12;16(1):171. doi: 10.1038/s41419-025-07446-x (PMC11903774; doi:10.1038/s41419-025-07446-x)
Supplement: Supplementary file 1 — Supplementary Figure legends [file 41419_2025_7446_MOESM1_ESM.docx]

**Supplementary Figures**

**Figure S1**

A. Gating strategies for TIM-1^+^ Bregs in the spleen, heart, and blood. B. Immunohistochemistry analysis TIM-1 levels in the sham, IRI, and IRI+RMT1-10 groups.

**Figure S2**

A. The percentage of CD4^+^T cells and CD8^+^ T cells in different groups were determined using Flow cytometry. B. The number of Treg cells in the sham, IRI, and IRI+RMT1-10 groups were determined from spleen, heart, and blood samples using Flow cytometry. C. Flow cytometry analysis of the number of Treg cells from the spleen, heart, and blood in the three groups (IRI, IRI+RMT1-10, and Anti-CD20+IRI+RMT1-10).
