# Supplementary figures and images for: The effect of TIM1+ Breg cells in liver ischemia-reperfusion injury
Source: Cell Death Dis. 2025 Mar 12;16(1):171. doi: 10.1038/s41419-025-07446-x (PMC11903774; doi:10.1038/s41419-025-07446-x)

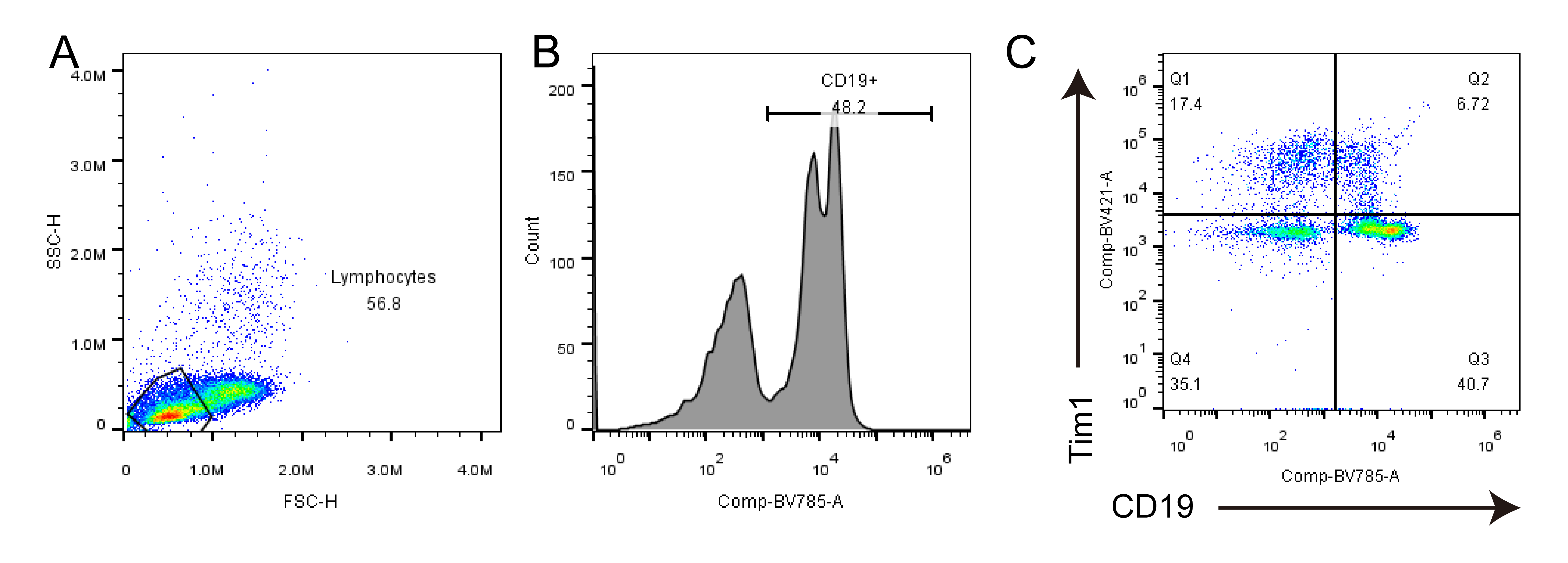

Supplement: Supplementary file 2 — Supplement Figure 1 [file 41419_2025_7446_MOESM2_ESM.tif]

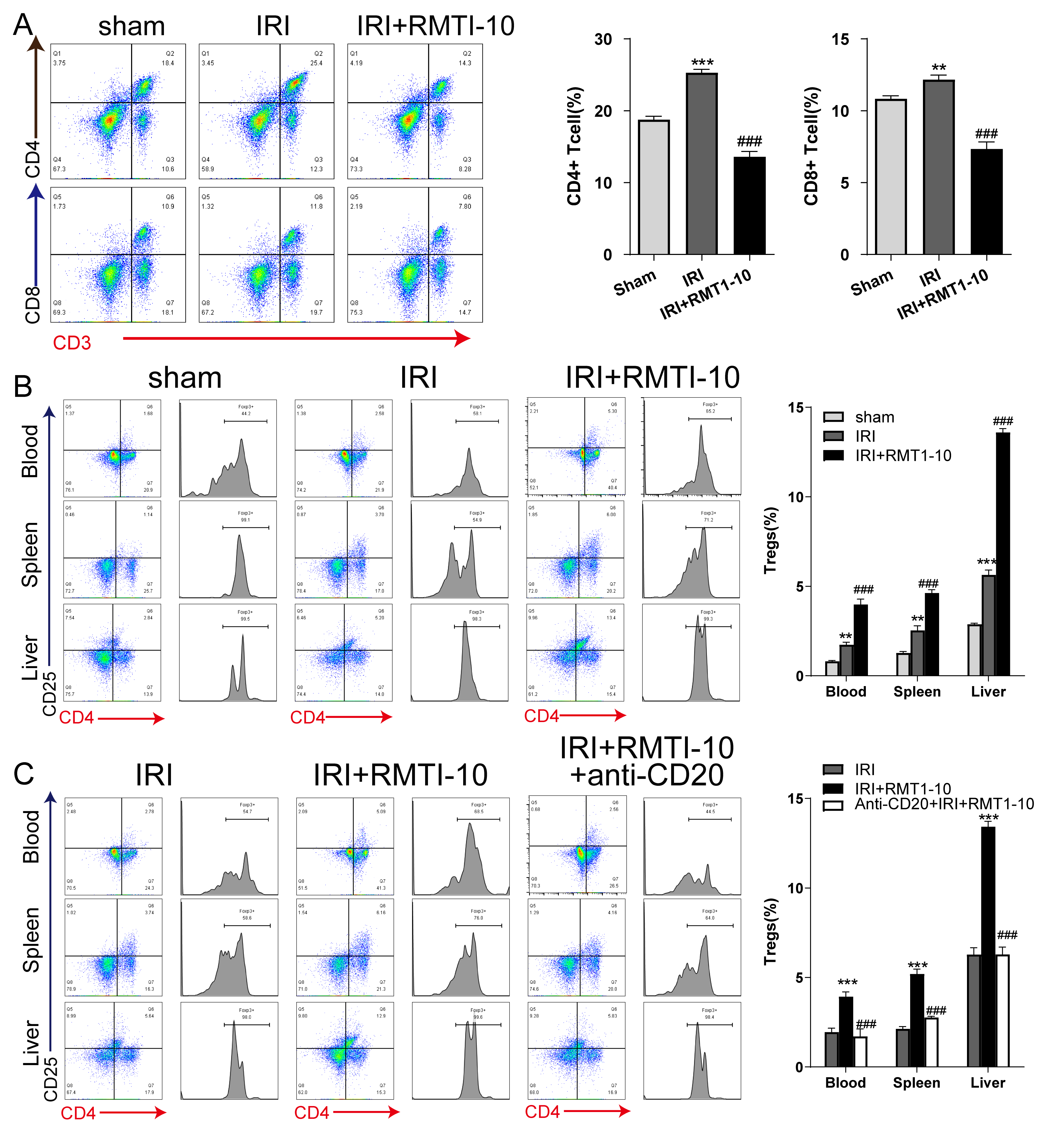

Supplement: Supplementary file 3 — Supplement Figure 2 [file 41419_2025_7446_MOESM3_ESM.tif]

Figure 7

IKKα


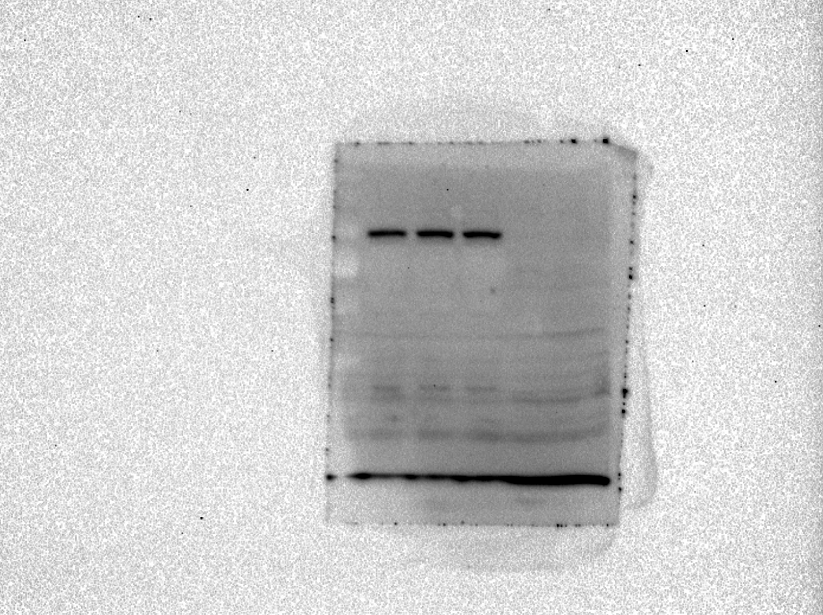


IKKβ


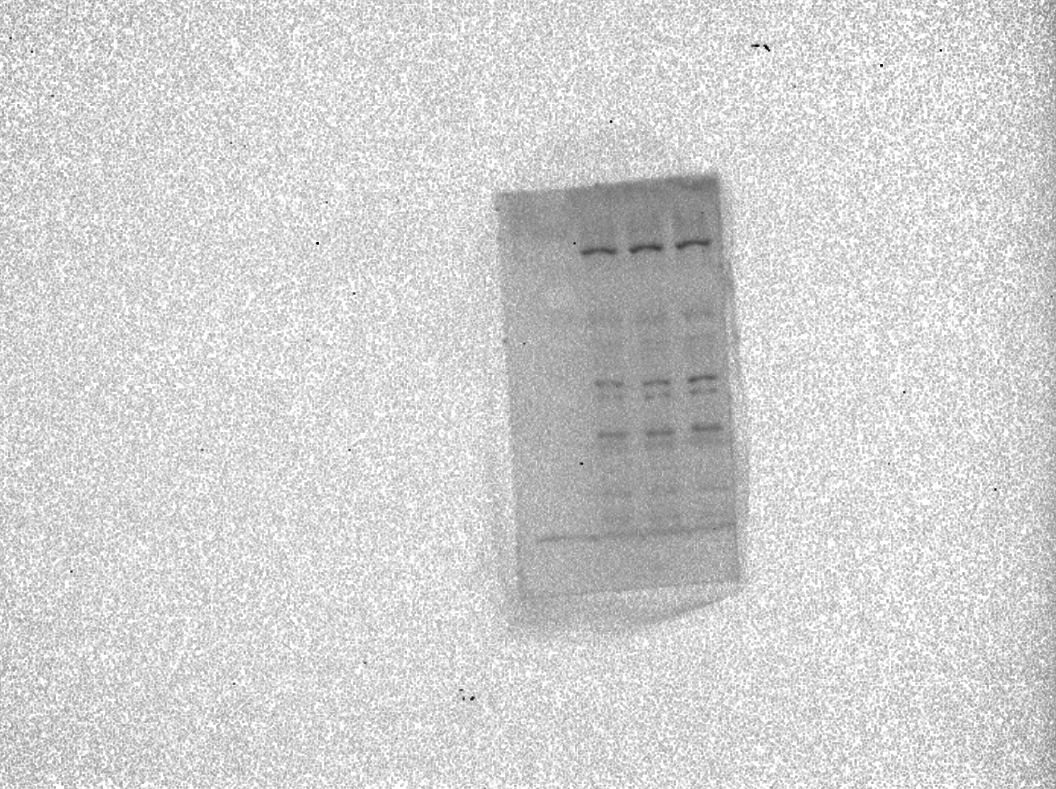


p-IKKα/β


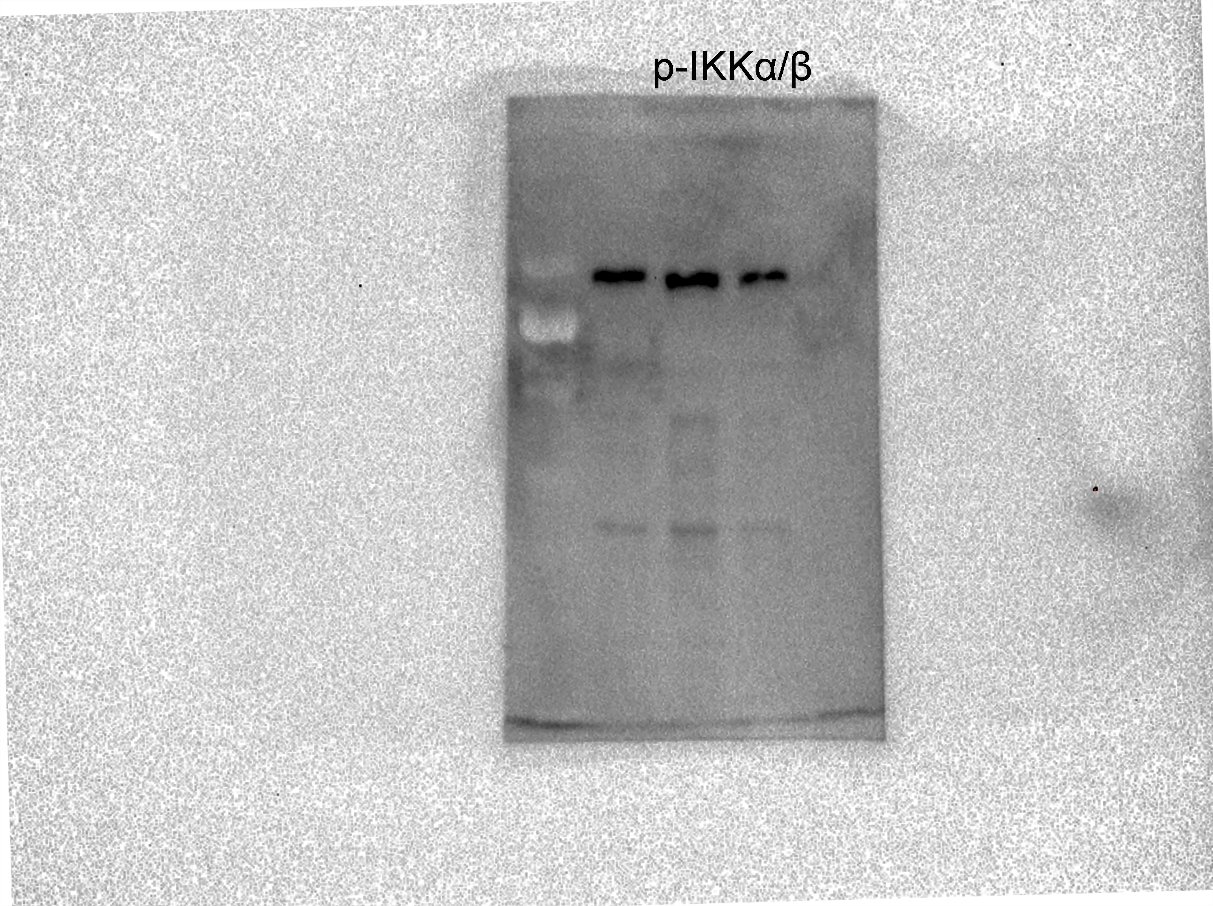


IκBα


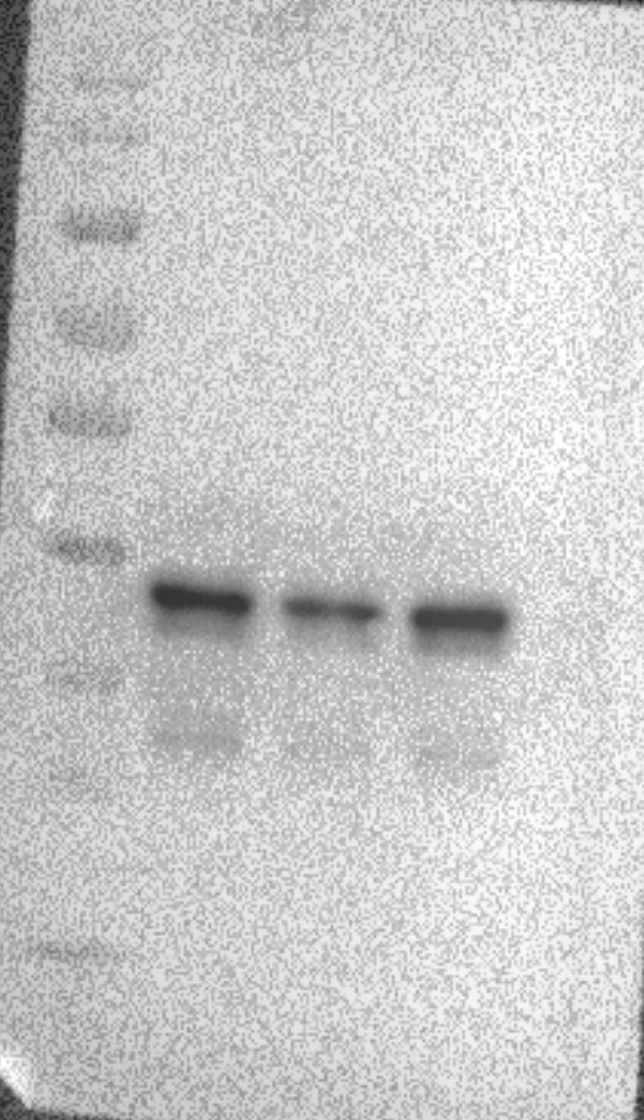


p- IκBα


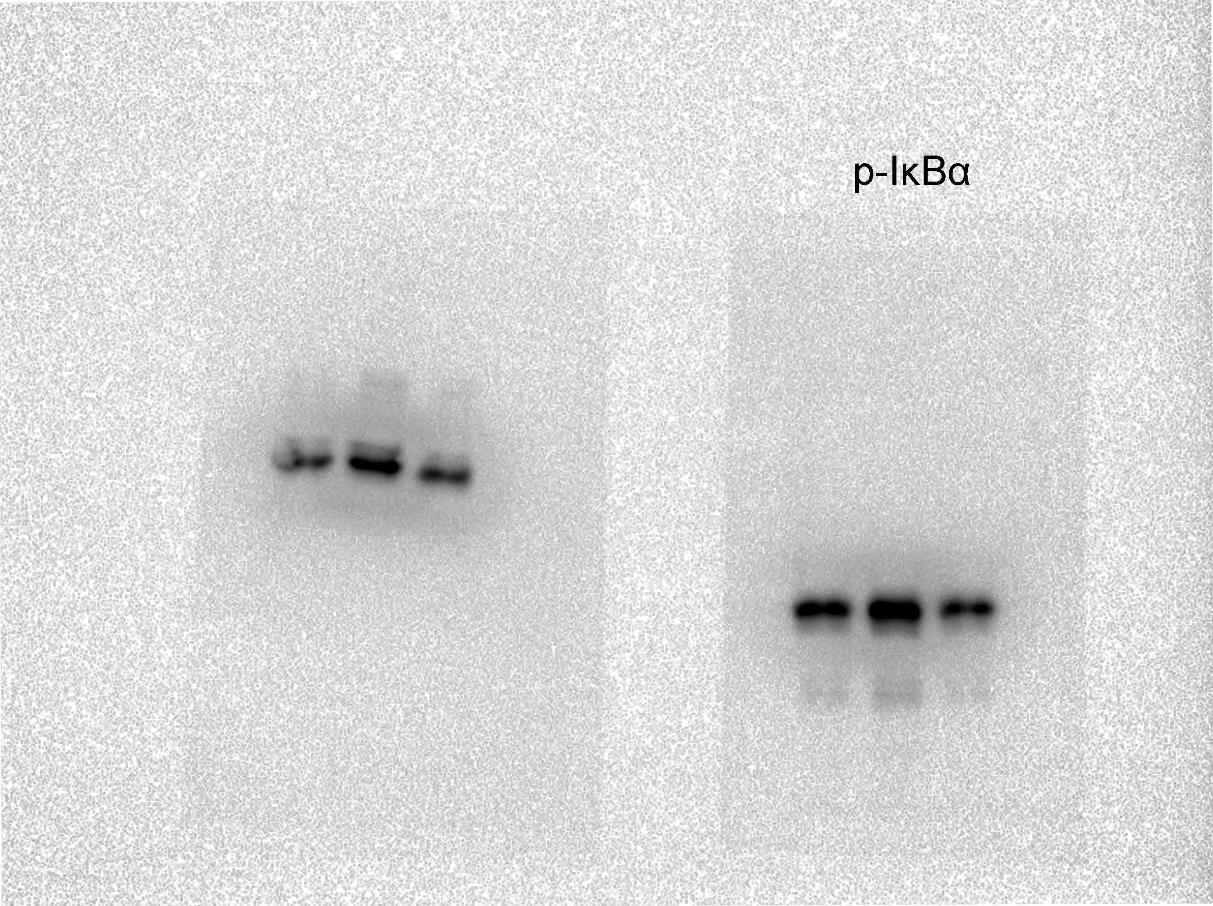
 B

β-catin


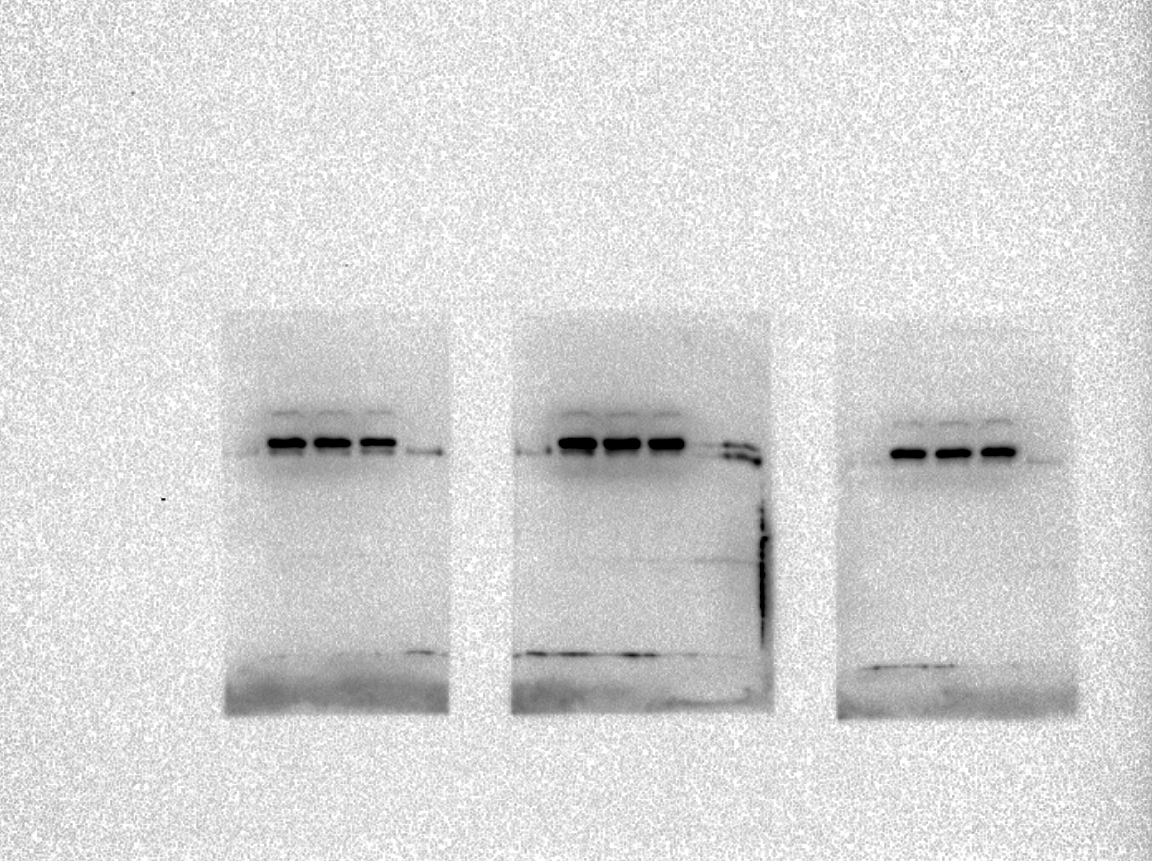


NF-κB


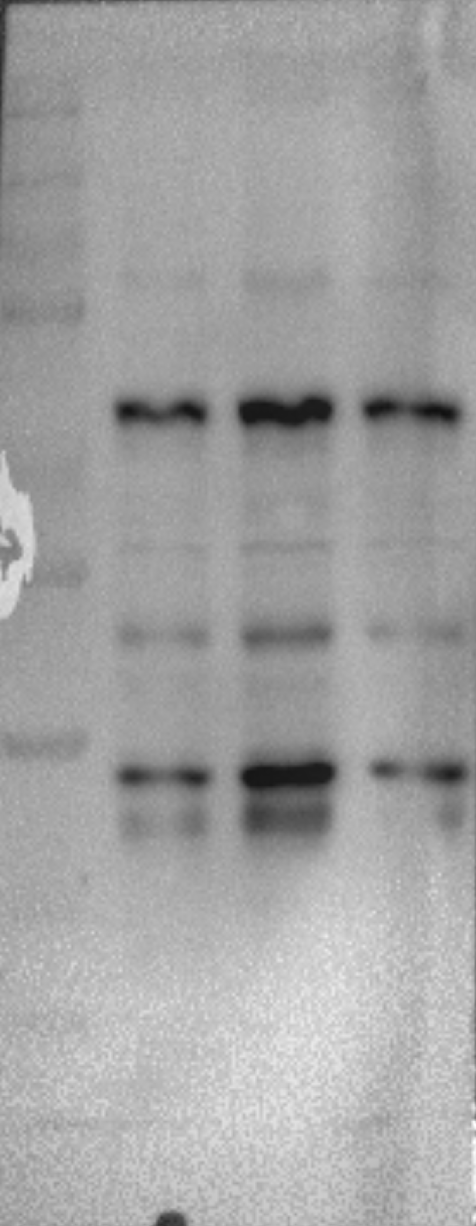

Supplement: Supplementary file 4 — Original WB [file 41419_2025_7446_MOESM4_ESM.docx]
